# Endothelial-specific Loss of IFT88 Promotes Endothelial-to-Mesenchymal Transition and Exacerbates Bleomycin-induced Pulmonary Fibrosis

**DOI:** 10.1038/s41598-020-61292-9

**Published:** 2020-03-11

**Authors:** Shweta Singh, Mohamed Adam, Pratiek N. Matkar, Antoinette Bugyei-Twum, Jean-Francois Desjardins, Hao H. Chen, Hien Nguyen, Hannah Bazinet, David Michels, Zongyi Liu, Elizabeth Mebrahtu, Lillian Esene, Jameela Joseph, Mehroz Ehsan, Mohammad Qadura, Kim A. Connelly, Howard Leong-Poi, Krishna K. Singh

**Affiliations:** 1 Department of Chemical and Biochemical Engineering, University of Western Ontario, London, ON N6A 5C1 Canada; 2 Department of Medical Biophysics, Schulich School of Medicine and Dentistry, University of Western Ontario, London, ON N6A 5C1 Canada; 3 Division of Cardiology, Keenan Research Centre for Biomedical Science and Li Ka Shing Knowledge Institute of St. Michael’s Hospital, Toronto, ON M5B 1W8 Canada; 4 Vascular Surgery, Keenan Research Centre for Biomedical Science and Li Ka Shing Knowledge Institute of St. Michael’s Hospital, Toronto, ON M5B 1W8 Canada; 5 Institute of Medical Science, University of Toronto, Toronto, ON M5S 1A1 Canada; 6 Department of Pharmacology and Toxicology, University of Toronto, Toronto, ON M5S 1A1 Canada; 7 Departments of Surgery, University of Toronto, Toronto, ON M5S 1A1 Canada; 8 Anatomy and Cell Biology, Schulich School of Medicine and Dentistry, University of Western Ontario, London, ON N6A 5C1 Canada; 9 Department of Biology, University of Western Ontario, London, ON N6A 5C1 Canada

**Keywords:** Mechanisms of disease, Heart failure

## Abstract

Intraflagellar transport protein 88 (Ift88) is required for ciliogenesis and shear stress-induced dissolution of cilia in embryonic endothelial cells coincides with endothelial-to-mesenchymal transition (EndMT) in the developing heart. EndMT is also suggested to underlie heart and lung fibrosis, however, the mechanism linking endothelial Ift88, its effect on EndMT and organ fibrosis remains mainly unexplored. We silenced *Ift88* in endothelial cells (ECs) *in vitro* and generated endothelial cell-specific Ift88-knockout mice (Ift88^endo^) *in vivo* to evaluate EndMT and its contribution towards organ fibrosis, respectively. *Ift88-*silencing in ECs led to mesenchymal cells-like changes in endothelial cells. The expression level of the endothelial markers (CD31, Tie-2 and VE-cadherin) were significantly reduced with a concomitant increase in the expression level of mesenchymal markers (αSMA, N-Cadherin and FSP-1) in *Ift88*-silenced ECs. Increased EndMT was associated with increased expression of profibrotic *Collagen I* expression and increased proliferation in *Ift88*-silenced ECs. Loss of Ift88 in ECs was further associated with increased expression of Sonic Hedgehog signaling effectors. *In vivo*, endothelial cells isolated from the heart and lung of Ift88^endo^ mice demonstrated loss of Ift88 expression in the endothelium. The Ift88^endo^ mice were born in expected Mendelian ratios without any adverse cardiac phenotypes at baseline. Cardiac and pulmonary endothelial cells isolated from the Ift88^endo^ mice demonstrated signs of EndMT and bleomycin treatment exacerbated pulmonary fibrosis in Ift88^endo^ mice. Pressure overload stress in the form of aortic banding did not reveal a significant difference in cardiac fibrosis between Ift88^endo^ mice and control mice. Our findings demonstrate a novel association between endothelial cilia with EndMT and cell proliferation and also show that loss of endothelial cilia-associated increase in EndMT contributes specifically towards pulmonary fibrosis.

## Introduction

Primary cilia (singular: cilium) are short (1–3 μm) hair-like projections arising from the surface of almost every vertebrate cell. Cilia are not completely bound by the plasma membrane but they represent a spatially distinct compartment separated from the rest of the cell^1^. Cilia depend upon an evolutionarily conserved mechanism of a microtubule-based transport system called **i**ntra**f**lagellar **t**ransport (**IFT**) for its maintenance and function^2^. Deletion of intraflagellar transport 88 (Ift88) results in loss of cilia^3^. Endothelial cells (ECs) line the heart and blood vessels and, as a result, are constantly exposed to hemodynamic forces. The endothelium is very sensitive to fluctuations in these dynamic physical and chemical conditions and, under physiological conditions, responds accordingly by releasing autocrine and paracrine factors^4^. The differential EC response to constantly varying flow patterns requires accurate mechanotransduction and mechanosensing^4^. In adult vasculature, primary cilia are located at atherosclerotic-prone sites where the flow is oscillatory and slow^5^, and recently a protective role of endothelial cilia was demonstrated where the loss of endothelial cilia promoted endothelial dysfunction and atherosclerosis in ApoE^null^ mice^6^. It is important to note that physiologically endothelial cilia is not a permanent structure and embryonic ECs which are exposed to high shear stress, temporarily dissolve their cilia and this process coincides with transforming growth factor-β (Tgfβ)-induced endothelial-to-mesenchymal transition (EndMT)^7^. High shear stress associated dissolution of cilia and EndMT occurs during the formation the primordia of cardiac valves during heart development^7^. EndMT is marked by fibroblast-like morphology, induction of Tgfβ signaling, loss of EC markers like CD31, VE-Cadherin and Tie-2, and gain of mesenchymal markers like alpha-smooth muscle actin (αSMA) and N-Cadherin^8,9^.

Although EndMT is well-established as a crucial process in heart development, it has now been implicated in a variety of pathologies including organ fibrosis and cancer^4,8^. The etiologic factors initiating fibrotic disorders are diverse and in most cases remain obscure, however, the accumulation of activated myofibroblasts remains a vital determinant of the severity and rate of disease progression and their response to therapy, prognosis as well as to mortality^10^. Previous observations that EndMT may also contribute to the generation of pro-fibrotic cells both *in vitro*^11^ as well as *in vivo*^12^, multiple immunofluorescence and EC-lineage-tracing studies conducted during the developmental course of various animal models of tissue fibrosis have demonstrated that EndMT is a significant source of tissue myofibroblasts^13^. EndMT, as a critical step in pulmonary fibrosis, was elegantly demonstrated by Hashimoto *et al*., who exploited lineage analysis to trace the source of interstitial fibroblasts in a bleomycin-induced lung fibrosis and reported that approximately 16% of lung fibroblasts were of endothelial origin^10,14,15^. These studies were validated by lineage-tracing and the results subsequently confirmed by other laboratories^4,10^. Cardiac fibrosis also results from an excessive extracellular matrix deposition mediated by the recruitment of fibroblasts from various sources^16–18^. Nonetheless, the origin of these fibroblasts is not completely understood^19^. Traditionally, adult fibroblasts directly originate from embryonic mesenchymal cells and increase in number due to the proliferation of resident fibroblasts^17,19^. Although controversial, albeit different stages, EndMT has been implicated in cardiac fibrosis, in addition to the proliferation of resident fibroblasts^20,21^. However, in the setting of loss of endothelial cilia, the contribution of EndMT towards fibroblasts accumulation, and cardiac and pulmonary fibrosis remains unknown. Furthermore, endothelial dysfunction predicts future cardiovascular morbidity^22^. While endothelial-cardiomyocyte crosstalk is influential in cardiac physiology/pathology^23,24^, the role of endothelial cilia in the cardiac remodeling caused by increased EndMT, either in development or pathogenesis, remains unclear.

The overarching goal of this study is to determine whether permanent loss of endothelial cilia induces EndMT and whether loss of cilia-associated increased EndMT exacerbates the progression of cardiac and pulmonary fibrosis. We hypothesize that an intact ciliary machinery plays an essential role in limiting EndMT and that loss of endothelial cilia results in impaired endothelial function and increased cardiac and pulmonary fibrosis. Our data demonstrate that loss of endothelial Ift88 leads to EndMT and increased proliferation *in vitro*, and exacerbates bleomycin-induced pulmonary fibrosis *in vivo*. Our data also indicates that loss of endothelial cilia associated EndMT does not contribute towards pressure-overload induced cardiac fibrosis, supporting studies where EndMT was demonstrated not to participate in cardiac fibrosis^18^.

## Results

### Loss of Ift88 induced marked morphological and ultrastructural changes, EndMT, increased proliferation and migration in ECs *in vitro*

**Figure 1:**
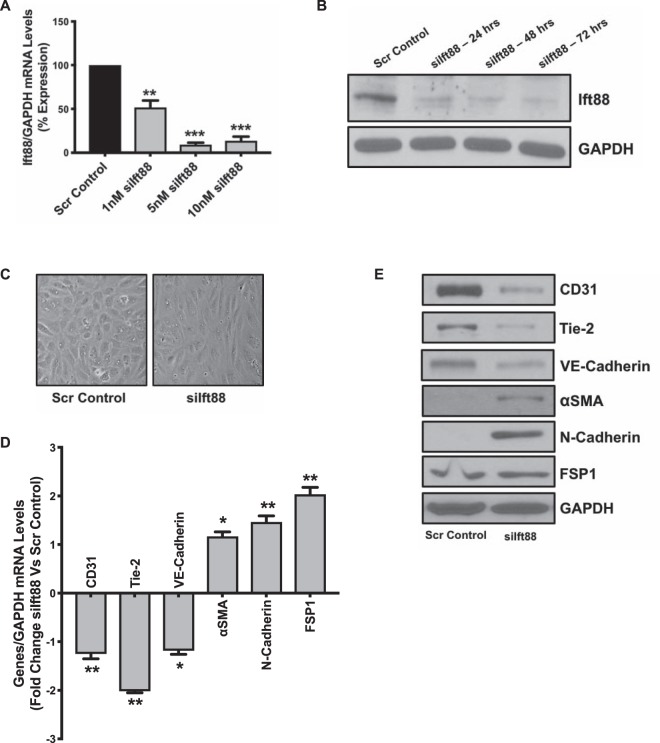
Loss of Ift88 induced marked morphological changes and EndMT in HUVECs. **(A)** Cultured HUVECs were transfected with either siIft88 or scrambled control. RT-PCR revealed successful silencing of Ift88 (~80% reduction) at 24 h. **(B)** Western blotting confirmed reduced Ift88 in Ift88-silenced HUVECs in a time-dependent manner. GAPDH was used as a loading control. **(C)** Scrambled control-transfected HUVECs, cultured on a two-dimensional plate, formed a confluent monolayer with the typical endothelial ‘cobblestone’ morphology (left panel). Ift88 silencing resulted in marked morphological changes whereby HUVECs took on an enlarged spindle-shaped appearance with smooth surfaces (right panel). Magnification (10×). **(D,E)** HUVECs were transfected with scrambled control or siIft88. Total RNA and protein were extracted at 24 h and 48 h, respectively. Differential **(D)** transcript data presented as a fold change to the scrambled control and **(E)** protein levels of key endothelial and mesenchymal markers are presented. GAPDH was used as a loading control. *p < 0.05, **p < 0.01 and ***p < 0.001 vs. corresponding scrambled control. *n* = 3–4 in triplicates.

We used siRNA technology to knock down *Ift88* expression in HUVECs. The cells were divided into three groups: 1. non-transfected, 2. scrambled control-transfected, and 3. siIft88-transfected ECs. The non-transfected cells were indistinguishable from the scrambled control-transfected cells therefore results from the non-transfected group is not presented. Silencing of Ift88 was optimized using three different doses (1, 5 and 10 nM) of siIft88. Quantitative PCR (qPCR) and immunoblot analysis confirmed successful silencing as evident by reduced *Ift88* expression at the transcript (Fig. 1A) and protein levels (Fig. 1B) in ECs transfected with 5 nM of siIft88 24, 48, and 72 hours post-transfection. Particularly, *Ift88*-silenced ECs demonstrated transition from the distinctive cobblestone-like morphology to an enlarged spindle-shaped fibroblast-like appearance indicative of EndMT in the *siIft88*-silenced ECs (Fig. 1C). Next, to evaluate EndMT, we examined the expression levels of endothelial (CD31, Tie-2 and VE-Cadherin) and mesenchymal [α-smooth muscle actin (αSMA), N-Cadherin and Fibroblast-specific Protein 1 (FSP1)] markers at the transcript and protein levels in scrambled control- and *siIft88*-transfected ECs. Our EndMT marker analysis data demonstrated a significant reduction in the expression level of endothelial markers CD31, Tie-2 and VE-Cadherin with a concomitant increase in the expression level of mesenchymal markers αSMA, N-Cadherin and FSP-1 at both the transcript (Fig. 1D) and protein (Fig. 1E) levels in the *siIft88*-transfected in comparison to scrambled control-transfected HUVECs.

**Figure 2:**
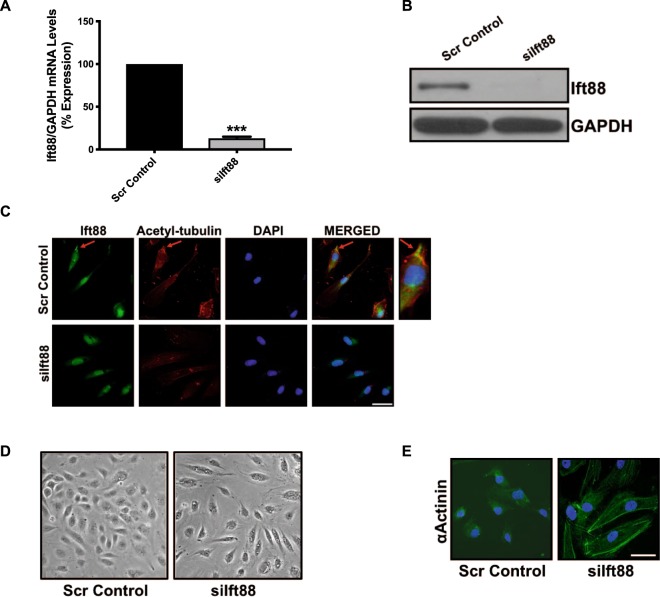
Loss of Ift88 led to the loss of cilia and induced marked morphological changes in HCAECs. **(A)** Cultured HCAECs were transfected with either siIft88 or scrambled control. RT-PCR revealed successful silencing of Ift88 (~80% reduction) at 24 h. **(B)** Western blotting confirmed lower Ift88 in HCAECs lacking Ift88. GAPDH was used as a loading control. **(C)** Immunofluorescent micrographs demonstrating siIft88-induced loss of cilia in HCAECs. Cilium is visualized by acetylated-tubulin staining (red). Ift88-positivity is indicated in green and nuclei were stained with DAPI (blue); scale bar = 10 µm. HCAEC with cilium is enlarged to clearly visualize the cilium in the merged picture (right panel). Cilium is indicated by a red arrow. **(D)** Scrambled control-transfected HCAECs, cultured on a two-dimensional plate, formed a confluent monolayer with the typical endothelial ‘cobblestone’ morphology (left panel). Ift88 silencing resulted in marked morphological changes whereby HCAECs took on an enlarged spindle-shaped appearance with smooth surfaces (right panel). Magnification (10×). **(E)** Immunofluorescent micrographs demonstrating cytoskeletal protein re-organization in HCAECs following Ift88 silencing. α-actinin positivity is indicated in green and nuclei were stained with DAPI (blue); scale bar = 5 µm. ***p < 0.001 vs. corresponding scrambled control. n = 3–4 in triplicates.

**Figure 3:**
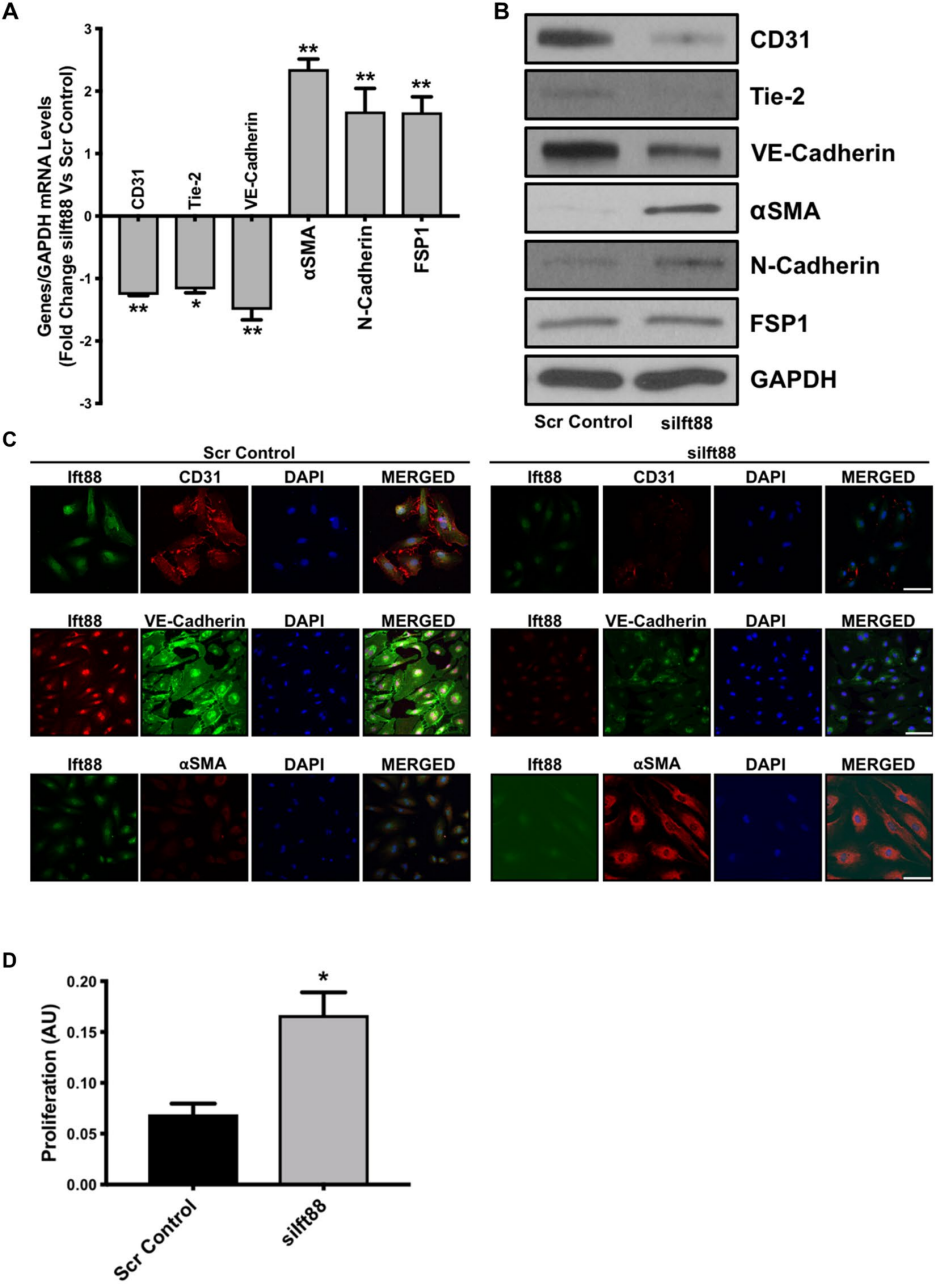
Loss of Ift88 induced EndMT in HCAECs. HCAECs were transfected with scrambled control or siIft88. Total RNA and protein were extracted at 24 h and 48 h, respectively. Differential **(A)** transcript (qPCR) data presented as a fold change to the scrambled control and **(B)** protein (western blotting) levels of key endothelial and mesenchymal markers, as well as **(C)** immunofluorescence for CD31 (red)/Ift88 (green), VE-Cadherin (green)/Ift88 (red) and αSMA (red)/Ift88 (green) staining in scramble control- and siIft88-transfected HCAECs indicate EndMT with Ift88 silencing. Nuclei were stained with DAPI (blue). Micrographs are representative images of HCAECs taken 72 h post-transfection; scale bar = 10 µm. **(D)** Loss of Ift88-induced cell proliferation in HCAECs following 24-hrs of silencing. *p < 0.05 and **p < 0.01 vs. corresponding scrambled control. *n* = 3–4 in triplicates.

**Figure 4:**
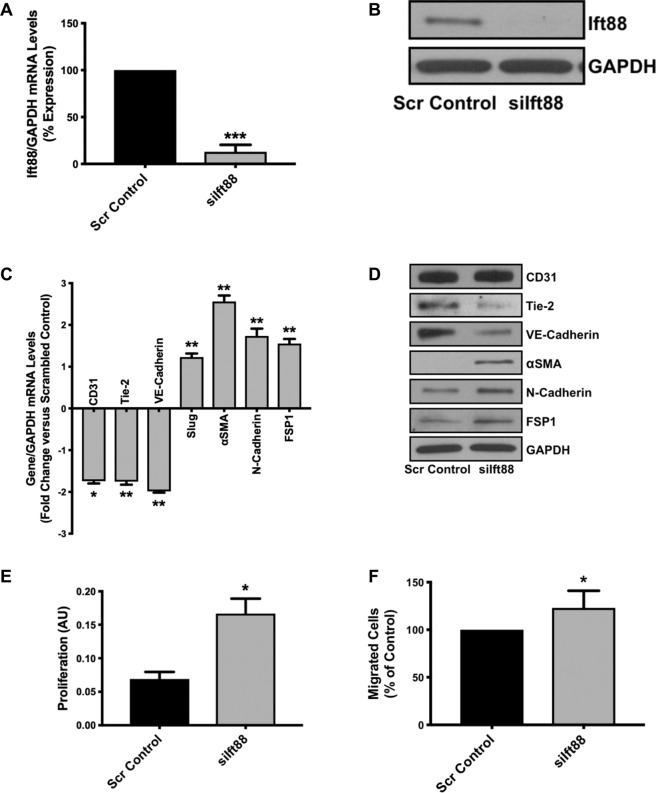
Loss of Ift88 caused increased EndMT, proliferation and migration in HPAECs. Cultured HPAECs were transfected with either siIft88 or scrambled control. Successful silencing of Ift88 at 24 h demonstrated by qPCR **(A)** and by immunoblotting **(B)** for Ift88 in HPAECs. GAPDH was used as a loading control. HPAECs were transfected with scrambled control or siIft88. Total RNA and protein were extracted at 24 h and 48 h, respectively. Differential **(C)** transcript (qPCR) data presented as a fold change to the scrambled control and **(D)** protein (Immunoblotting) levels of key endothelial and mesenchymal markers in *Ift88*-silenced and control HPAECs. **(E)** Loss of Ift88-induced cell proliferation and **(F)** migration in HPAECs, measured 24-hrs post-transfection. *p < 0.05 and **p < 0.01 vs. corresponding scrambled control. *n* = 3–4 in triplicates.

Furthermore, to confirm that loss of Ift88-associated EndMT is not specific to HUVECs we silenced *Ift88* in HCAECs and HPAECs and evaluated the phenotype. We were successfully able to silence *Ift88* in HCAECs at the transcript (Fig. 2A) and at the protein level (Fig. 2B). The *siIft88*-silenced and scrambled control-transfected endothelial cells were immunostained with antibodies for Ift88 and also for acetylated-tubulin to observe the cilia. Loss of Ift88 caused the loss of cilia in *siIft88*-transfected HCAECs (Fig. 2C). We also observed the loss of cobblestone-like morphology and gain of enlarged spindle-shaped pattern in the HCAECs (Fig. 2D). These changes were accompanied by an elevation in expression and organized arrangements for mesenchymal cell-like cytoskeletal protein *α*-actinin (Fig. 2E). Next, to evaluate EndMT, we examined the expression levels of endothelial (CD31, Tie-2 and VE-Cadherin) and mesenchymal (αSMA, N-Cadherin and FSP1) markers at the transcript and protein levels in scrambled control- and *siIft88*-transfected HCAECs. Our EndMT marker analysis data demonstrated a significant reduction in the expression level of endothelial markers CD31, Tie-2 and VE-Cadherin with a concomitant significant increase in the expression level of mesenchymal markers αSMA, N-Cadherin and FSP-1 at both transcript (Fig. 3A) and protein (Fig. 3B) levels in the *siIft88*-transfected in comparison to scrambled control-transfected HCAECs. EndMT was also confirmed by immunofluorescence for Ift88, VE-Cadherin and αSMA in siIft88- and scrambled control-transfected HCAECs. Immunofluorescence data further confirmed EndMT with reduced CD31 and VE-Cadherin and increased αSMA expression in the Ift88-silenced in comparison to the scrambled control-transfected HCAECs (Fig. 3C). In humans, endothelial cells undergoing EndMT demonstrate higher proliferation and migration^25,26^. To determine if the loss of Ift88-associated EndMT is also associated with increased proliferation and migration, we evaluated proliferation in scramble control- and *siIft88*-transfected HCAECs. In line with previous findings, our data demonstrated an increased proliferation in the Ift88-silenced in comparison to the scrambled-control transfected HCAECs (Fig. 3D). Similar to HCAECs, we were also able to silence *Ift88* at the transcript and the protein level in HPAECs (Fig. 4A,B). Loss of Ift88 in HPAECs led to EndMT switching at the transcript and the protein level (Fig. 4C,D), as well as increased proliferation and migration (Fig. 4E,F). Overall, our data demonstrate that loss of endothelial cilia-associated EndMT and increased proliferation is not specific to an endothelial cell type.

### Ift88 Loss does not activate TGFβ and Wnt/β-catenin signaling but Up-regulates sonic hedgehog (Shh) signaling in ECs *in vitro*

**Figure 5:**
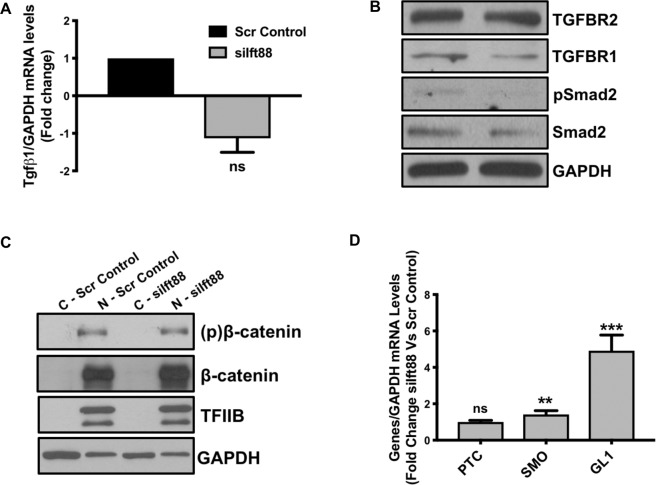
Loss of Ift88 does not affect Tgfβ and Wnt/β-catenin signaling but up-regulates Sonic Hedgehog (SHH) signaling. HCAECs were transfected with siIft88 or scrambled control and total RNA and protein were extracted at 24 h and 48 h, respectively. **(A)** Quantitative PCR for TGFβ1 at the transcript level and **(B)** Western blot for TGFBR1, TGFBR2, pSmad2 and Smad2 show no effect of Ift88 silencing. **(C)** HCAECs were transfected with siIft88 or scrambled control and cytoplasmic and nuclear protein were extracted at 48 h to perform immunoblot for β-catenin, (p) β-catenin. TFIIB and GAPDH were used as loading controls for nuclear and cytoplasmic protein, respectively. **(D)** Loss of Ift88 was associated with increased SMO and GLI1 in endothelial cells indicating enhanced SHH signaling. **p < 0.01 and ***p < 0.0001 vs. corresponding scrambled control group. n = 3–4 in triplicates.

TGFβ-signaling is the main inducer of EndMT^27^. Cilia regulates sonic hedgehog (shh) and Wnt/β-Catenin signaling^28–30^. Wnt/β-Catenin interacts with Tgfβ-signaling, and loss of cilia is associated with an increased translocation of β-Catenin to the nucleus that is also known to participate in the induction of EndMT^31^. Therefore, we evaluated the effect of loss of Ift88 or cilia on these three Tgfβ-, Wnt/β-Catenin- and Shh-signaling in ECs. To our surprise, we did not observe any significant changes in the expression level of ligand Tgfβ1, receptors TGFBR1 and TGFBR2 and effectors such as Smad2 of Tgfβ-signaling and Tgfβ-target gene CTGF in the *Ift88-*silenced ECs, when compared with scrambled control-transfected ECs (Fig. 5A,B; Suppl. Fig. 1A,B). Similar data were obtained across HCAEC (Fig. 5A,B), HUVEC (Suppl. Fig. 1A) and HPAECs (data not shown). Additionally, we also measured the expression levels of Tgfβ1, TGFBR1 and TGFBR2 in the MLECs isolated from WT and Ift88^endo^ mice and did not observe any significant difference between the groups (Suppl. Fig. 1C–E). Next, we treated scrambled control and siIft88-tranfected ECs with human recombinant Tgfβ1 and measured the expression level of EndMT markers and Ift88. Our data show a clear induction of EndMT and reduction in Ift88 in the control ECs but Tgfβ1-treatment had no additive effect on the EndMT marker expression in the Ift88-silenced ECs (Suppl. Fig. 2A,B). Moreover, we also did not observe any significant changes in the expression level of β-Catenin in the siIft88-silenced in comparison to scrambled control-transfected ECs. Given that β-Catenin translocates to the nucleus for its activity, we measured the translocation of β-Catenin from the cytoplasm to the nucleus, which appears not to be affected by the loss of Ift88 in ECs (Fig. 5C). Lastly, Shh is known to be regulated by cilia and loss of cilia is associated with reduced Shh signaling^32^. However, we observed that the receptor for PTC that plays an obligate negative regulatory role in the Shh-signaling pathway was unaffected, but the mediators of Shh-signaling, such as SMO and zinc-finger transcription factor GL1, were significantly up-regulated in the Ift88-silenced ECs (Fig. 5D).

### Endothelial cell-specific loss of Ift88-associated increase in EndMT does not exacerbate pressure overload-induced cardiac fibrosis *in vivo*

**Figure 6:**
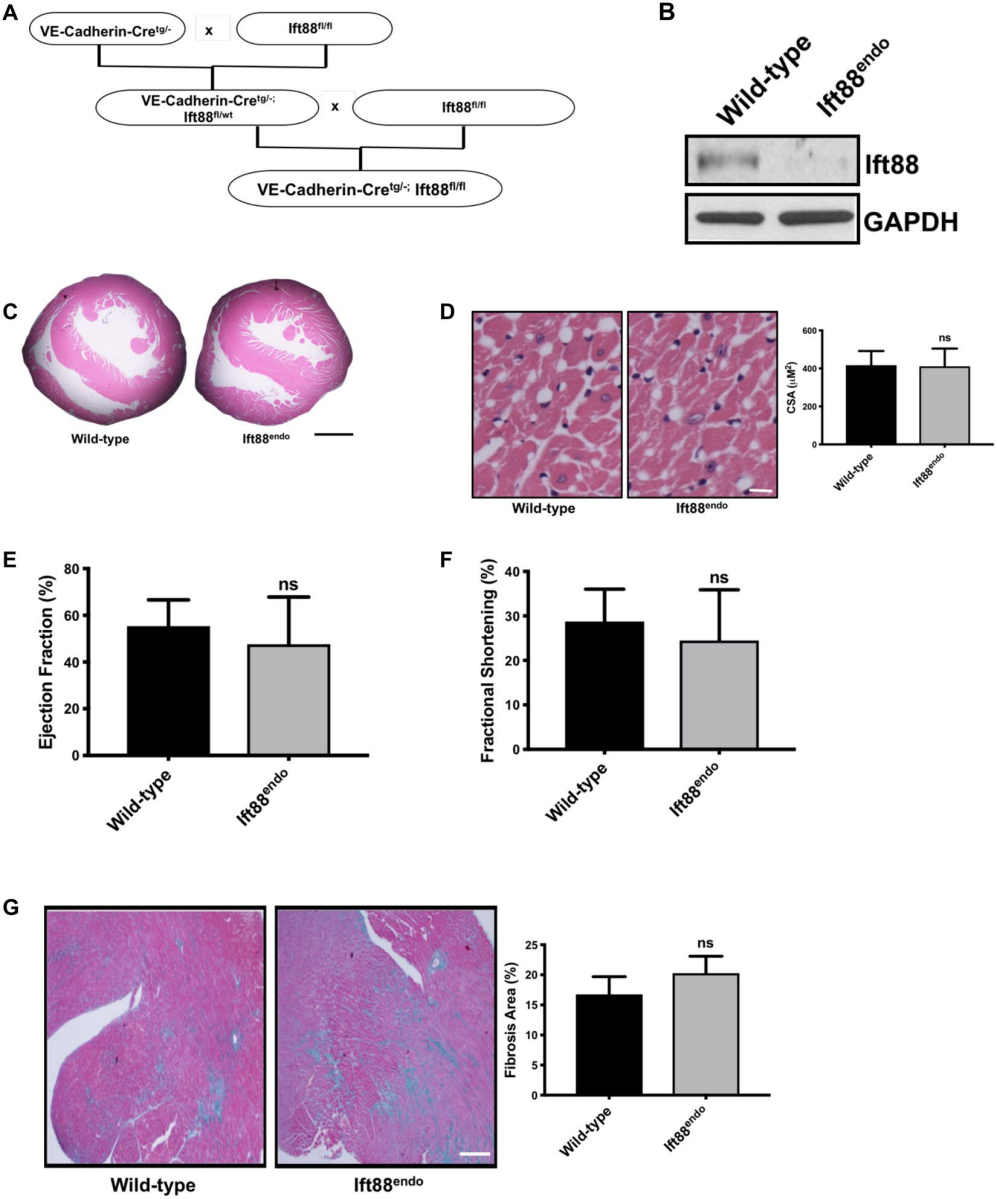
Endothelial-specific loss of Ift88 did not induce adverse phenotype at baseline and did not exacerbate cardiac fibrosis following TAC. (A) To generate endothelial-specific Ift88 knockout (Ift88^endo^) mice, mice homozygous for the floxed Ift88 allele (Ift88^fl/fl^) were crossed with mice expressing Cre-recombinase under the control of the VE-Cadherin promoter (VE-Cadherin-Cre^tg^/^−^) to generate VE-Cadherin-Cre^tg/−^;Ift887^fl/wt^ mice. VE-Cadherin-Cre^tg/−^;Ift88^fl/wt^ mice were subsequently crossed with Ift887^fl/fl^ mice to generate VE-Cadherin-Cre^tg/−^;Ift88^fl/fl^ (Ift88^endo^) and littermate control VE-Cadherin-Cre^tg/−^;Ift88^wt/wt^ mice as depicted in the figure. **(B)** Total protein isolated from the cardiac endothelial cells of Ift88^endo^ and WT control littermates showed reduced Ift88 expression by immunoblot in Ift88^endo^ mice. GAPDH was used as a loading control. **(C**,**D)** Ift88^endo^ mice exhibited no obvious cardiac phenotypes as demonstrated by H&E staining of the baseline transverse cardiac sections **(C)** and by measuring the myocytes cross-sectional area (CSA) **(D)**. ns = non-significant vs. corresponding WT control group. Scale bar = 500 µm, n = 6/group, age = 12 weeks. **(E**,**F)** Stress in the form of TAC was induced to Ift88^endo^ and WT control littermates (n = 12/group, age = 12 weeks). Cardiac function was measured after 6 weeks of TAC via echocardiography and **(E)** ejection fraction (EF) and **(F)** fractional shortening (FS) were measured. **(G)** Following echocardiography, hearts were collected, sectioned and stained for total collagen content by Masson’s Trichrome. The bar graph represents the collagen content quantification (n = 12/group, ns = non-significant).

In order to extend our *in vitro* findings to an *in vivo* setting, endothelial cell-specific Ift88 knockout (Ift88^endo^) mice were generated using *Cre-LoxP* technology and were characterized (Fig. 6A). Successful deletion of Ift88 specifically in the cardiac ECs (CECs) was performed by isolating CECs, where CECs from the Ift88^endo^ mice revealed a virtual absence of Ift88 protein in comparison to CECs from WT control littermates (Fig. 6B). These Ift88^endo^ mice were born in the expected Mendelian ratios, exhibited no obvious cardiovascular phenotypes or changes in blood pressure, and displayed similar cardiac structure and function in accordance with a previous publication^6^. A histomorphometric assessment was performed on *in situ* fixed and H&E-stained hearts. Similar baseline cardiac morphologies and structures were found in Ift88^endo^ mice and their WT littermate controls (Fig. 6C,D). There were no differences in the left ventricular inner (2.31 ± 0.42 mm vs 2.42 ± 0.92 mm, WT vs Ift88^endo^, p = n.s.) and outer (3.87 ± 0.85 mm vs 3.91 ± 0.77 mm, WT vs Ift88^endo^, p = n.s) diameter, septal wall thickness (1.04 ± 0.13 mm vs 0.99 ± 0.26 mm, WT vs Ift88^endo^, p = n.s), and average cross sectional area of cardiomyocytes between the groups (Fig. 6C,D). Echocardiographic measurements demonstrated an indistinguishable cardiac function between Ift88^endo^ mice and their WT littermate controls at 8–12 weeks of age (Table 1). The transverse aortic constriction (TAC) model is a widely employed model to study pressure overload-induced cardiac fibrosis and heart failure. Accordingly, we induced TAC in the Ift88^endo^ mice and the WT control littermates and measured cardiac function and fibrosis 6-weeks after TAC. To our surprise, our data demonstrated that endothelial-specific loss of Ift88 did not exacerbate cardiac dysfunction as measured by echocardiography (Fig. 6E,F) and fibrosis as measured by staining for collagen deposition between Ift88^endo^ mice and the WT control littermates (Fig. 6G).

**Table 1:** Echocardiographic assessments demonstrate a similar cardiac function at baseline between control and Ift88^endo^ mice.

Cardiac Parameters	WT Control Littermates	Ift88^endo^
Heart rate (bpm)	433 ± 29	441 ± 31
LV end-diastolic dimension (cm)	0.317 ± 0.028	0.325 ± 0.032
LV end-systolic dimension (cm)	0.269 ± 0.039	0.275 ± 0.41
LV ejection fraction (%)	63 ± 12.1	61 ± 13.6
LV fractional shortening (%)	28.1 ± 8.32	29.1 ± 7.1

Data presented as mean ± SD (n = 6/group).

### Endothelial cell-specific loss of Ift88 induces EndMT and exacerbates bleomycin (BLM)-induced pulmonary fibrosis *in vivo*

**Figure 7:**
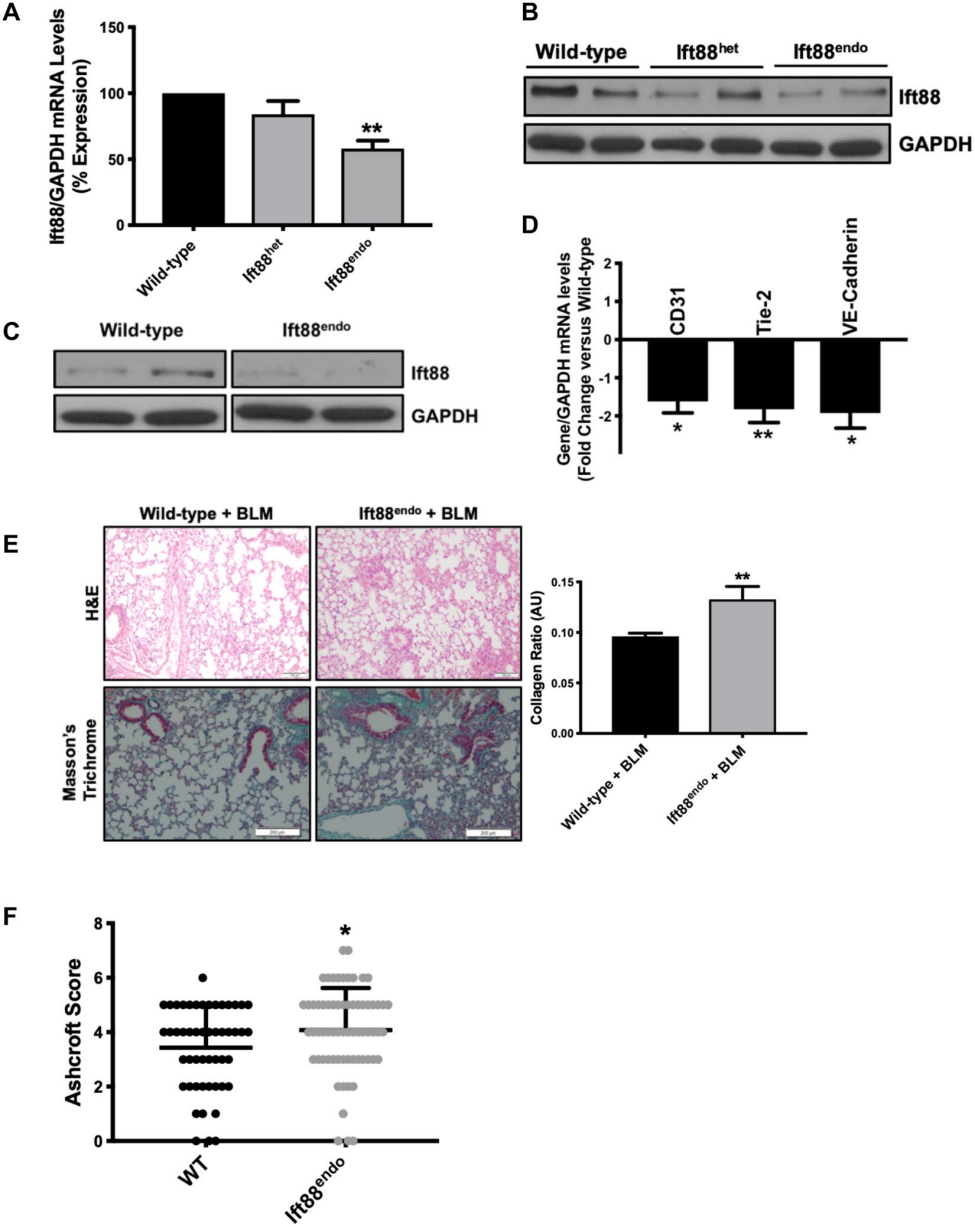
Endothelial-specific loss of Ift88 induced EndMT at baseline and exacerbated bleomycin-induced pulmonary fibrosis. Total RNA and protein were isolated from the lungs of Ift88^endo^ and WT control littermates and qPCR and immunoblot of Ift88 was performed. Data show reduced Ift88 expression by qPCR **(A)** immunoblot **(B)** in Ift88^endo^ mice. GAPDH was used as a loading control. **(C,D)** Protein and RNA were isolated from the Mouse lung endothelial cells (MLECs) of Ift88^endo^ mice and control littermates and immunoblot for Ift88 and qPCR for endothelial markers were performed, respectively. Data showed complete loss of Ift88 at protein level in the MLECs of Ift88^endo^ mice **(C)**. qPCR demonstrated reduced endothelial markers expression in the MLECs of Ift88^endo^ mice **(D)**. **(E)** Following 21 days of bleomycin treatment to the Ift88^endo^ mice and control littermates, lungs were collected, sectioned and stained for H&E and total collagen content by Masson’s Trichrome. The bar graph represents the collagen content quantification. **(F)** Ashcroft score of pulmonary fibrosis. (n = 12/group, **p < 0.001 vs. corresponding wild-type control littermates).

To further confirm the successful deletion of Ift88, we isolated RNA and protein from the lung tissues of the wild-type, endothelial cell-specific Ift88 heterozygous knockout mice (Ift88^het^) and Ift88^endo^ mice and measured Ift88 expression. The Ift88 transcript and Ift88 protein analysis demonstrated reduced expression of Ift88 in the lung (Fig. 7A,B). To evaluate the deletion of Ift88 specifically in the mouse lung endothelial cells (MLECs), MLECs were isolated from the WT and IFT88^endo^ mice and Ift88 was measured. Ift88 transcript and the protein data confirmed a complete absence of Ift88 in the Ift88^endo^ MLECs at the protein levels (Fig. 7C). Next, to validate the loss of Ift88-associated EndMT in HPAECs in the MLECs isolated from the Ift88^endo^, all the EndMT markers were evaluated. The transcript data performed for the endothelial markers demonstrated a significant reduction (Fig. 7D), as observed in the *Ift88*-silenced ECs. To evaluate the potential translational implications of our *in vitro* data, bleomycin (BLM) was instilled in the lungs of the wild-type and Ift88^endo^ mice and lung fibrosis was evaluated. BLM induces variable pulmonary fibrosis based on the strain and genetic background of the mice, however, WT and IFT88^end^ mice utilized in the current study were of mixed background and BLM is shown to induce pulmonary fibrosis in these mice^33–35^. We observed profound alterations in the pulmonary microanatomy of BLM-treated Ift88^endo^ when compared with similarly treated wild-type mice. These differences included increased alveolar septal thickening and infiltration of the parenchyma by inflammatory cells with a resultant increase in pulmonary fibrosis and collagen content 21 days after BLM instillation in the Ift88^endo^ mice (Fig. 7E). These data were further confirmed by evaluating the Ashcroft score, which also demonstrated BLM-induced exacerbated pulmonary fibrosis in Ift88^endo^ in comparison to WT mice (Fig. 7F).

## Discussion

Organ fibrosis represents the final common pathway through which various ischemic, hemodynamic, inflammatory, and genotoxic risk factors lead to organ failure. Amongst the several mechanisms that have been suggested to underlie organ fibrosis, increasing data points toward a critical and previously underappreciated role of endothelial-to-mesenchymal transition (EndMT) as a source of fibroblasts. These data have generated considerable translational enthusiasm since they imply that molecular and/or cellular targeting of EndMT may serve to limit aberrant fibroblastic flux and, through this mechanism, reduce the genesis of organs, such as cardiac and pulmonary fibrosis. Our overall aim was to evaluate novel mechanisms governing EndMT, EndMT-mediated aberrant remodeling and organ fibrosis. Specifically, we hypothesized that endothelial primary cilia play a critical role in the process of EndMT, and that loss of endothelial primary cilial assembly is a fundamental step through which endothelial cells acquire a pathologic mesenchymal phenotype that in turn culminates in cardiac and pulmonary fibrosis, and organ failure. We investigated this hypothesis by: (i) silencing the cilial assembly gene *Intraflagellar transport 88 (Ift88)* in the endothelial cells and then by evaluating EndMT, and (ii) by conducting comprehensive studies in mice that have the essential ciliary assembly gene conditionally knocked out in endothelial cells.

Successful *Ift88* knockdown *via* RNA silencing (siRNA) in ECs led to the loss of cilia (Figs. 1A,B and 2A–C). Our studies further indicated that loss of cilia promoted EndMT and was accompanied by marked morphological and ultrastructural changes whereby ECs transitioned from the typical “cobblestone-like” morphology to an enlarged spindle-shaped, smooth surfaced “fibroblast-like” morphology (Figs. 1C and 2D). Notably, at the subcellular level, *Ift88*-silenced ECs exhibited cytoskeletal protein re-organization as confirmed by α-actinin staining (Fig. 2E). At the molecular level, disruption of Ift88 expression was associated with reduced expression of the EC markers CD31, Tie-2 and VE-Cadherin, and increased expression of the mesenchymal markers αSMA, N-Cadherin and fibroblast-specific protein (FSP1) (Figs. 1D,E, 3A–C and 4C,D). Changes observed with loss of Ift88 in HUVECs were mirrored in Human Coronary Artery ECs (HCAECs) and Human Pulmonary Artery ECs (HPAECs) (Figs. 1–4).

Deletion of Ift88 results in the loss of cilia^3^. Cilia amplify the cytoskeletal strain and regulate endothelial function^36^. Furthermore, the endocardial cushions in the embryonic heart, which are exposed to high shear stress, are marked by the temporary dissolution of cilia in embryonic ECs^37^. These ECs with dissolved cilia undergo Tgfβ-driven EndMT^7^. EndMT is thought to be initiated by inductive signals like Tgfβs and β-Catenin from the myocardium^7,38–41^. Although EndMT is crucial in heart development, it has also been implicated in a wide variety of pathologies namely cancer and organ fibrosis^4,8,33^. A recent study demonstrates that loss of endothelial cilia promotes endothelial dysfunction and atherosclerosis in ApoE^null^ mice^42^. Given that EndMT can be defined as a gain of mesenchymal cell function, but at the same time, it can also be looked as a loss of endothelial function. Therefore, loss of endothelial cilia-associated increased atherosclerosis in ApoE^null^ mice might have been due to EndMT-mediated loss of endothelial function. Cilia mainly regulate sonic hedgehog (Shh) and Wnt/β-Catenin signaling^28–30^, where Wnt/β-Catenin interacts with Tgfβ-signaling, and loss of cilia are associated with increased translocation of β-Catenin to the nucleus that induces EndMT^31^. The dissolution of primary cilia is known to induce Tgfβ signaling and EndMT in mice embryonic endothelial cells^43^. The Shh-signaling pathway is known to play an important role during the developmental process^44^, however, Shh-signaling is not known to be associated with the process of EndMT. Therefore, we postulated that EC-specific loss of Ift88 or cilia may result in aberrant Wnt/β-Catenin and Tgfβ signaling, leading to increased EndMT in adult human endothelial cells. To our surprise, the ligand for Tgfβ-signaling Tgfβ1, Tgfβ receptors; TGFBR1 and TGFBR2, and the effectors: Smad2 and pSmad2 were not significantly affected in *Ift88*-silenced ECs (Fig. 5A,B). To further confirm that loss of Ift88/cilia associated effect on Tgfβ-signaling is not HCAECs-specific, we measured the expression of TGFBR1, TGFBR2, Smad2 and pSMad2 in *Ift88*-silenced HUVECs and observed similar results, as these Tgfβ-signaling associated molecules were also not affected in *Ift88*-silenced HUVECs (Suppl. Fig. 1A,B). We then measured the expression level of Tgfβ1, TGFBR1 and TGFBR2 in the MLECs isolated from adult WT and Ift88^endo^ mice, which also did not show any significant change in these molecules (Suppl. Fig. 1C–E). All of these evaluated Tgfβ-related molecules appear to be non-significant in Ift88-deficient ECs in comparison to control ECs, indicating that loss of endothelial cilia associated activation of Tgfβ-signaling is differentially regulated in mice embryonic endothelial cells *versus* adult endothelial cells, potentially due to higher plasticity of embryonic endothelial cells. To evaluate any possible correlation between Ift88 and Tgfβ-signaling in adult human ECs, we then treated control and Ift88-silenced ECs with recombinant human Tgfβ1 and measured the extent of EndMT marker expression and the expression level of Ift88. Tgfβ1-treatment significantly induced EndMT in scrambled control-transfected ECs, as previously reported^33,45^, but interestingly, had no additive effect on the extent of EndMT in the Ift88-silenced ECs (Suppl. Fig. 2A). Tgfβ1-induced EndMT coincided with reduced expression of Ift88, indicating an inverse relationship between EndMT and Ift88/cilia in response to Tgfβ1 (Suppl. Fig. 2B). We also did not observe any significant change in the expression or activity of β-Catenin in the *Ift88*-silenced ECs as expected in comparison to controls (Fig. 5C). Therefore, we looked at the effect of loss of Ift88 on Shh-signaling and evaluated the expression level of PTC, SMO, and GL1. We observed a significantly up-regulated SMO and GL1 in the *Ift88*-silenced in comparison to scrambled control-transfected ECs (Fig. 5D). This is a novel finding as Shh-signaling is not known to be associated with EndMT. However, further studies are currently underway to understand the contribution of the upregulation of SMO and GL1 in relation to loss of Ift88/cilia in ECs, and also the association, if any, of Shh signaling with EndMT.

Cardiac fibrosis is mediated by the recruitment of fibroblasts, and accordingly, there has been an immense interest to gain a better understanding of the molecular underpinnings through which fibroblasts accumulate since they may offer clues for potential translational evaluation and treatment of heart failure. Although the source of fibroblasts in cardiac fibrosis is controversial, it is expected to be multifactorial. Recent data indicate that cardiac fibrosis is associated with the emergence of fibroblasts originating from ECs and that EndMT, an event that occurs during the formation of the atrioventricular cushion in the embryonic heart, may also play an important, albeit pathological role, in the post-natal state^18,19^. Furthermore, increased mesenchymal cell proliferation and migration is associated with EndMT and tissue fibrosis^25,46^. Transforming growth factor-β1 (Tgfβ1) induces ECs to undergo EndMT, whereas Bone Morphogenetic Protein 7 (BMP-7), which preserves the endothelial phenotype, significantly inhibits EndMT and the progression of cardiac fibrosis in mouse models of pressure overload^19^. Primary cilia are cellular protrusions that serve as mechanosensors for fluid flow and also render ECs prone to shear-induced EndMT in embryonic endothelial cells of mice^43^. Therefore, we hypothesized that an intact ciliary machinery plays a fundamental role in limiting aberrant EndMT and that loss of endothelial cilial assembly unleashes EndMT, exacerbating cardiac fibrosis. Accordingly, and also to extend our *in vitro* findings to an *in vivo* setting, endothelial cell-specific Ift88 knockout (Ift88^endo^) mice were generated using *Cre-LoxP* technology and were characterized (Fig. 6A–D). These mice were born in expected Mendelian ratios, exhibited no obvious cardiovascular phenotypes or changes in blood pressure, and displayed similar cardiac structure and function (Fig. 6C,D) in accordance with a previous publication^6^. Despite normal endothelial and vascular architecture, CECs from these mice exhibited reduced expression levels of CD31 and VE-Cadherin. Interestingly, in spite of these differences, structurally and functionally, the hearts of Ift88^endo^ mice were indistinguishable from those of their wild-type littermate controls under baseline conditions. Furthermore, to understand the implication of our *in vitro* findings and also to delineate the contribution of EndMT towards cardiac fibrosis, we used the transverse aortic constriction (TAC) model, which is a widely employed animal model to study cardiac fibrosis. Although we observed the signs of EndMT in the cardiac ECs, the extent of cardiac fibrosis was not different between the Ift88^endo^ and the control littermates (Fig. 6G). Similarly, we did not observe any difference in the cardiac functions when measured post-TAC *via* echocardiography (Fig. 6E, F). Our data indicate that the loss of endothelial cilia-associated EndMT is not a significant source of fibroblast exacerbating cardiac fibrosis in Ift88^endo^ mice after TAC, which supports a previous finding that the cardiac resident fibroblasts but not the EndMT-related fibroblasts contribute to the genesis of pressure overload-associated cardiac fibrosis^18,19^.

EndMT is elegantly demonstrated as a critical step in the progression of pulmonary fibrosis by lineage-tracing the source of interstitial fibroblasts in bleomycin-induced lung fibrosis, and approximately 16% of lung fibroblasts were found to be of endothelial origin^10,14,15^. We isolated MLECs from the lungs of Ift88^endo^ and the wild-type control littermates. We then confirmed the successful deletion of Ift88 and EndMT-like switching as all the endothelial markers were significantly reduced (Fig. 7C,D). Given the confirmed contribution of EndMT in the exacerbation of bleomycin-induced EndMT, we hypothesized that loss of endothelial cilia-associated increase in EndMT will exacerbate bleomycin-induced pulmonary fibrosis. To understand, the role of loss of endothelial cilia-associated EndMT in the progression of bleomycin-induced pulmonary fibrosis, we instilled bleomycin to the lungs of Ift88^endo^ and the wild-type control littermates. Upon instilling bleomycin into the lungs, we observed increased alveolar septal thickening, infiltration of the parenchyma by inflammatory cells, and increase in bronchocentric collagen content in the fibrotic lungs of Ift88^endo^ mice in comparison to the wild-type control littermates (Fig. 7E). BLM-induced exacerbated pulmonary fibrosis in Ift88^endo^ in comparison to WT mice was also confirmed by an increased Ashcroft score in the lung sections of BLM-treated ft88^endo^ mice (Fig. 7F). These results indicate that loss of endothelial cilia-associated increase in EndMT in the lungs of Ift88^endo^ mice exacerbated pulmonary fibrosis, which supports our hypothesis. Next, we aim to delineate the exact contribution of cilia-associated EndMT towards exacerbated pulmonary fibrosis utilizing the lineage-tracing technique. However, a major determinant of negative prognosis in pulmonary fibrosis is the degree of new reactive fibroblasts recruited to the lungs, thus therapeutic methods targeting the sources of fibroblasts are effective approaches in order to improve the fibrotic condition and its complication. Our current study presents novel findings of the mechanism through which lung endothelial cells could possibly contribute to pulmonary fibrosis. We demonstrated *in vitro* and *in vivo* that lungs endothelial cells undergoing EndMT could serve to be a critical source of fibroblasts and an inducer of EndMT is the loss of endothelial primary cilia. Future studies are, thus, warranted to investigate approaches to prevent the loss of endothelial cilia-associated EndMT or EndMT *per se* and the related signaling mechanisms in the development of pulmonary fibrosis.

## Materials and Methods

### Cell culture and *Ift88* silencing

Human Umbilical Vein ECs **(**HUVECs, Lonza), Human Coronary Artery ECs **(**HCAECs, Lonza) and Human Pulmonary Artery ECs (HPAECs, Lonza) were grown in EC growth medium-2 (EGM™-2 Bulletkit™; Lonza) containing growth factors or MCDB 131 (Gibco) supplemented with serum and antibiotics. siRNA-mediated *Ift88* gene knockdown studies were performed with 5 nM siIft88 or scrambled control (Ambion) and the Dharmafect-4 transfection reagent (Dharmacon) in accordance with the manufacturer’s instructions. Following 60–70% confluence, cells were starved overnight and then treated with 10 ng TGFβ1 (Santa Cruz Biotechnology) and the control groups were treated with the diluent. Cardiac primary endothelial cells were isolated using the protocol as described^47^.

### Proliferation and migration assay

HCAECs were seeded at a density of 1–1.5×10^4^ cells/well in 96-well plates, transfected with siIft88 or scrambled control. Cell proliferation was evaluated 24 hrs post-transfection using WST-8 Cell Proliferation Assay Kit (Cayman Chemicals) according to the manufacturer’s guidelines. Following 24 hrs of transfection with either siIft88 or scrambled control, HPAECs were re-seeded in a density of 2×10^5^ cells/well in the upper chamber of the Cyto-Select 24-well Cell Migration Fluorometric Assay system (Cell Biolabs, Inc). Cell migration was evaluated after 12-hrs of incubation according to the manufacturer’s instruction.

### Quantitative real-time PCR

Total RNA was extracted using Trizol® as instructed (Invitrogen). Complementary DNA (cDNA) was synthesized using the Quantitect kit (Qiagen). Quantitative polymerase chain reaction (qPCR) was performed using the ABI ViiA 7 Real-Time PCR System (Applied Biosystems). For the PCR reaction, SYBR® Select Master Mix or TaqMan® Gene Expression Assays (both Applied Biosystems) were mixed with forward and reverse primers for *Ift88, CD31, VE-Cadherin, Tie-2, αSMA*, *N-Cadherin, fibroblast-specific protein-1 (FSP1), Connective Tissue Growth factor (CTGF), TGFβ1, Patched (PTC;* Forward – 5′-CTC-ATA-TTT-GGG-GCC-TTC-G-3′; Reverse – 5′-TCT-CCA-ATC-TTC-TGG-CGA-GT-3′*), smoothened protein (SMO;* Forward – 5′-GG-CTC-CCA-GGA-GGA-AGC-3′; Reverse – 5′-GGT-CAT-TCT-CAC-ACT-TGG-GC-3′*), Glioma-associated oncogene homolog (GL1;* Forward – 5′-CCA-GCG-CCC-AGA-CAG-AG-3′; Reverse – 5′-GGC-TCG-CCA-TAG-CTA-CTG-AT-3′) and *GAPDH* according to the manufacturer’s recommendations and as described previously^33,48,49^.

### Immunoblot, immunofluorescence and immunohistochemistry

Transfected ECs were harvested 24, 48 and 72 hour post-transfection with either siIft88 or scrambled control. Total cell lysates were prepared in RIPA buffer (Sigma Aldrich) and protein was extracted and quantified. An Equal amount of proteins were loaded on sodium dodecyl sulfate (SDS) polyacrylamide gels for immunoblotting analysis. The primary antibodies were utilized at a dilution of 1:1000: Ift88 (Santa Cruz Biotechnology #84318), CD31 (Cell Signaling #3528), VE-Cadherin (Santa Cruz Biotechnology #6458), Tie2 (Santa Cruz Biotechnology #324), N-Cadherin (abcam #ab76057), FSP1 (Abnova #H00006275-M01), αSMA (abcam #ab5694), α-actinin (Cell Signaling #3134), TGFβ1 (abcam #ab9758), SMAD2 (Cell Signaling #3122), pSMAD2 (Cell Signaling #3101), β-catenin (Cell Signaling #8480), phospho-β-catenin (Cell Signaling #9561), and GAPDH (Millipore #MAB374). The blot was developed using an enhanced chemiluminescence substrate (SuperSignal^TM^, Life Technologies) and a ChemiDoc^TM^ imaging system (Bio-Rad). Densitometry (ImageJ software) was performed to quantify the band intensities. Immunofluorescence experiments were carried out in 4-chamber microscopy slides performed as previously described^33,48,49^. Immunofluorescence signals from Acetyl-tubulin, CD31, VE-Cadherin, αSMA, Ift88 and α-actinin staining were visualized with standard protocols 24 hours post-treatment. Fluorescent microscopy images were captured using the Zeiss LSM700 confocal microscope and ZEN imaging software was utilized for image processing. Hearts and lung tissues were perfused, harvested, and cleared of extraneous tissue before being fixed in O.C.T. compound (Tissue-Tek®) for cryosectioning. Sections were stained with hematoxylin and eosin (H&E) or Masson’s trichrome. The severity of pulmonary fibrosis was quantified by Ashcroft scoring system^50^.

### Generation of endothelial cell-specific Ift88 knock-out mice and aortic banding

EC-specific **Ift88** knock-out (Ift88^endo^) mice were generated via crossing floxed **Ift88** mice (Ift88^flox/flox^ mice) with VE-cadherin Cre transgenic mice^6,33^. Generation and the characterization of EC-specific *Ift88* knock-out mice has been described previously^6^. A single dose of BLM (0.0015 units/g body weight, Sigma) was instilled endotracheally on day 0- to 20-week-old mice^51^. All animal-related experiments and procedures were reviewed and approved by the St. Michael’s Hospital Animal Care Committee, and all methods were performed in accordance with the relevant guidelines and regulations.

### Statistical analysis

Student’s ***T-***test was utilized to compare the means of two groups. Differences between multiple means were calculated by analysis of variance, and when overall differences were detected, individual means were compared post-hoc with the Bonferroni’s test. A ***p-***value <0.05 was accepted as statistical significance. Data are expressed as the mean ± SD.

## Supplementary information

Supplementary Figures.
