# Supplementary material for: Endothelial-specific Loss of IFT88 Promotes Endothelial-to-Mesenchymal Transition and Exacerbates Bleomycin-induced Pulmonary Fibrosis
Source: Sci Rep. 2020 Mar 11;10:4466. doi: 10.1038/s41598-020-61292-9 (PMC7066128; doi:10.1038/s41598-020-61292-9)
Supplement: Supplementary file 1 — Supplementary Figures. [file 41598_2020_61292_MOESM1_ESM.pdf]

Endothelial-specific Loss of IFT88 Promotes Endothelial-to-Mesenchymal Transition and Exacerbates Bleomycin-induced Pulmonary Fibrosis

Shweta Singh[1], Mohamed Adam[3,5], Pratiek N. Matkar[3,5], Antoinette Bugyei-Twum[3,5], Jean-Francois Desjardins[3], Hao H. Chen[3,5], Hien Nguyen[2,8], Hannah Bazinet[2], David Michels[2], Zongyi Liu[2], Elizabeth Mebrahtu[2], Lillian Esene[2], Jameela Joseph[2,9], Mehroz Ehsan[2], Mohammad Qadura[4,5], Kim A. Connelly[3,5], Howard Leong-Poi[3,5], Krishna K. Singh[2,4,5,6,7,8†]

SUPPLEMENTARY FIGURE 1

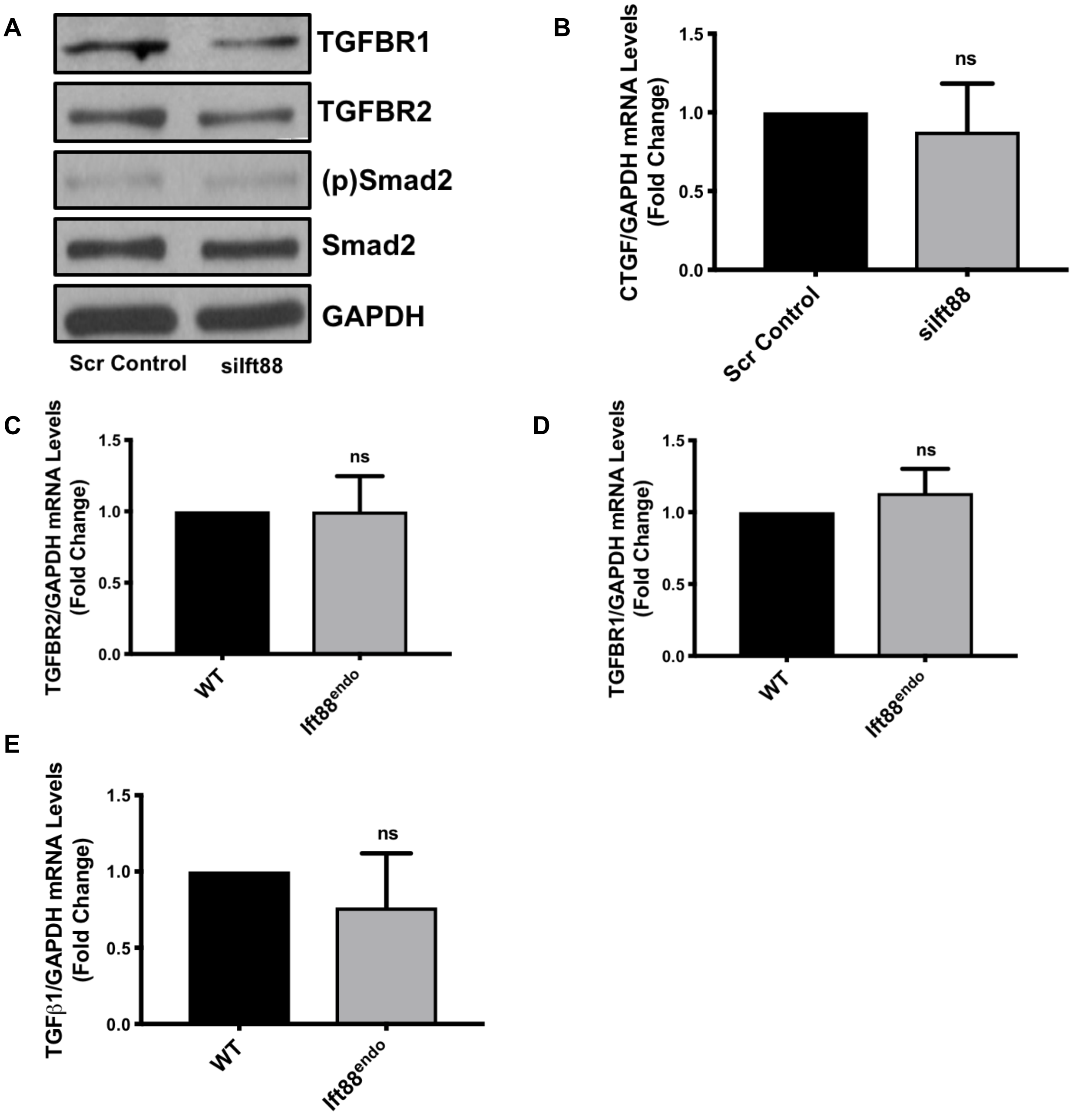

Supplementary Figure 1. Loss of Ift88 in adult ECs does not induce Tgfβ signaling. (A) Loss of Ift88 in HUVECs did not affect TGFBR1, TGFBR2 expression, and SMAD2 expression and activation. **(B)** qPCR performed for CTGF expression in scrambled control and siIFT88-transfected HPAECs. **(C-E)** Expression analysis of Tgfβ1, TGFBR1 and TGFBR2 in the MLECs of WT and $Ift88^{endo}$ mice. RNAs were isolated from MLECs obtained from of WT and $Ift88^{endo}$ mice and qPCR was performed for **(C)** Tgfβ1, **(D)** TGFBR1 and **(E)** TGFBR2. (n = 3-5/group, ns; non-significant).

A

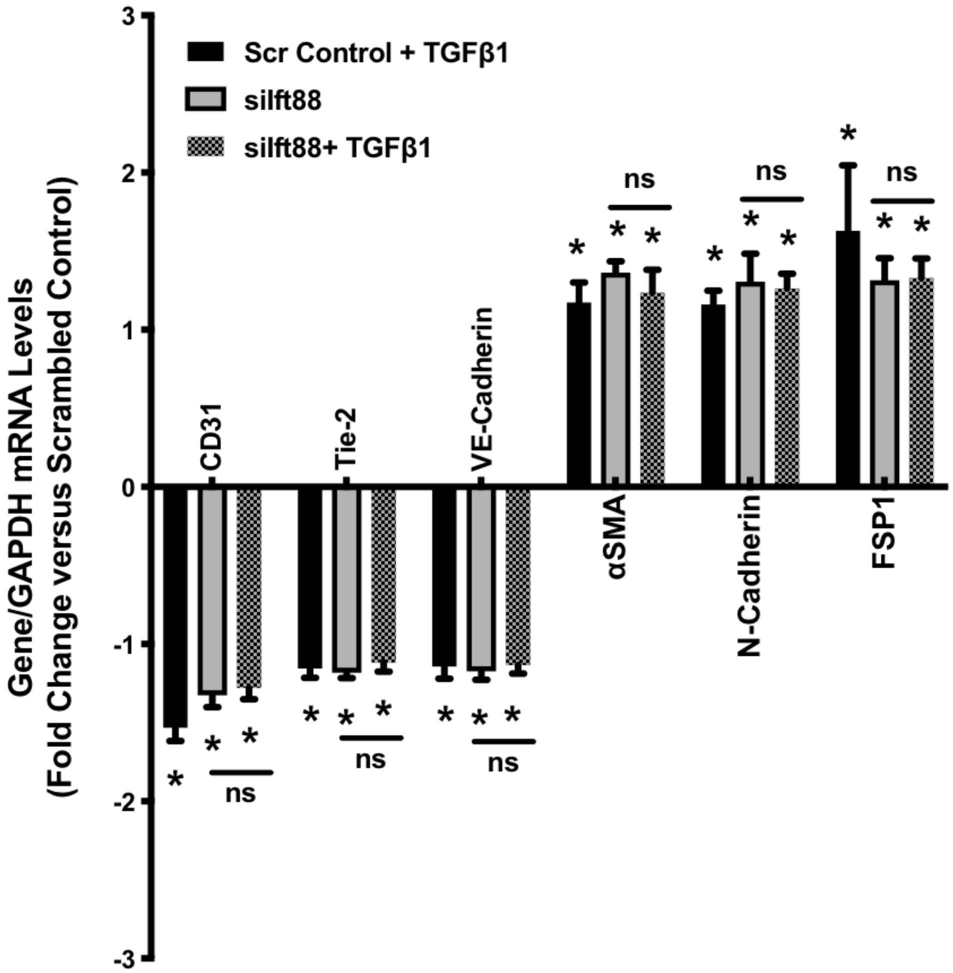

B

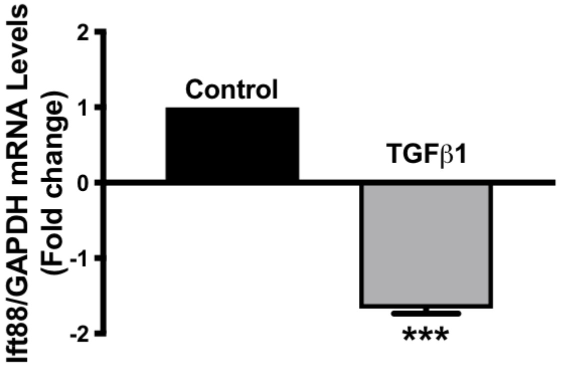

Supplementary Figure 2. Tgfβ1-treatment induced EndMT and reduced Ift88 expression in the control ECs but did not exacerbate loss of Ift88-associated EndMT in Ift88-silenced ECs. HPAECs were transfected with either scrambled control or siIft88 and after 48 hrs ECs were treated with Tgfβ1 (10ng/ml) for additional 24 hours of treatment and RNA were extracted to perform qPCR. qPCR data demonstrating **(A)** EndMT markers and **(B)** Ift88 expression. (n = 3-4 in triplicate, *,***$p<0.05$, 0.001 vs scrambled control).
